# 4-Cyanoindole-2′-deoxyribonucleoside as a Dual Fluorescence and Infrared Probe of DNA Structure and Dynamics

**DOI:** 10.3390/molecules24030602

**Published:** 2019-02-08

**Authors:** Ismail A. Ahmed, Arusha Acharyya, Christina M. Eng, Jeffrey M. Rodgers, William F. DeGrado, Hyunil Jo, Feng Gai

**Affiliations:** 1 Department of Biochemistry and Biophysics, University of Pennsylvania, Philadelphia, PA 19104, USA; aismail@pennmedicine.upenn.edu; 2 Department of Chemistry, University of Pennsylvania, Philadelphia, PA 19104, USA; arusha@sas.upenn.edu (A.A.); chrie@seas.upenn.edu (C.M.E.); jeffrey.rodgers@pennmedicine.upenn.edu (J.M.R.); 3 Department of Pharmaceutical Chemistry, University of California, San Francisco, CA 94158, USA; bill.degrado@ucsf.edu

**Keywords:** infrared, fluorescence, site-specific spectroscopic probe, 4-cyanoindole, unnatural nucleosides, DNA

## Abstract

Unnatural nucleosides possessing unique spectroscopic properties that mimic natural nucleobases in both size and chemical structure are ideally suited for spectroscopic measurements of DNA/RNA structure and dynamics in a site-specific manner. However, such unnatural nucleosides are scarce, which prompts us to explore the utility of a recently found unnatural nucleoside, 4-cyanoindole-2′-deoxyribonucleoside (4CNI-NS), as a site-specific spectroscopic probe of DNA. A recent study revealed that 4CNI-NS is a universal nucleobase that maintains the high fluorescence quantum yield of 4-cyanoindole and that among the four natural nucleobases, only guanine can significantly quench its fluorescence. Herein, we further show that the C≡N stretching frequency of 4CNI-NS is sensitive to the local environment, making it a useful site-specific infrared probe of oligonucleotides. In addition, we demonstrate that the fluorescence-quencher pair formed by 4CNI-NS and guanine can be used to quantitatively assess the binding affinity of a single-stranded DNA to the protein system of interest via fluorescence spectroscopy, among other applications. We believe that this fluorescence binding assay is especially useful as its potentiality allows high-throughput screening of DNA–protein interactions.

## 1. Introduction

**Figure 1:**
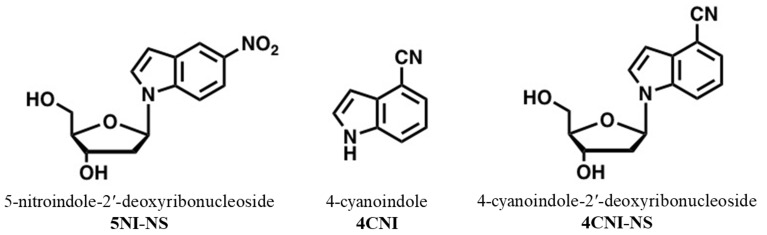
Structures of 5-nitroindole-2′-deoxyribonucleoside (5NI-NS), 4-cyanoindole (4CNI) and 4-cyanoindole-2′-deoxyribonucleoside (4CNI-NS).

Bio-macromolecules, including proteins and polynucleotides such as DNA and RNA, can adopt various complex structures that confer specific functions. Therefore, understanding the structure–function relationship of such bio-macromolecules has been the subject of numerous studies, many of which use infrared- and/or fluorescence-based spectroscopic techniques due to their sensitivity and convenience. However, intrinsic infrared (IR) and fluorescence signals of bio-macromolecules, while very useful, often provide only limited information about the system in question. Therefore, the past two decades have seen significant efforts in the development of extrinsic IR and fluorescence probes that can be used to site-specifically interrogate the structure, dynamics, and function of bio-macromolecules [1,2]. However, introducing an exogenous component into any bio-macromolecule will unavoidably perturb its structure (and consequently other properties). Therefore, many past studies have focused on identifying molecular moieties that not only afford the required spectroscopic attributes and can be easily incorporated into the bio-macromolecules of interest, but also minimally perturb their native structure. In this regard, the ideal extrinsic spectroscopic probes for protein (DNA) would be simple analogues of naturally-occurring amino acids (nucleosides). Indeed, a large body of work has been devoted to this idea, especially in the area of protein science. As a result, a large pool of unnatural amino acid-based (UAA-based) IR and fluorescence probes have been identified and utilized to study a wide range of problems in protein biochemistry and biophysics [3,4,5,6,7,8]. In comparison, however, little has been done in the development of minimally-perturbing, unnatural nucleoside-based spectroscopic probes. This is due, at least in part, to the fact that the fundamental building blocks of DNA involve only four nucleobases in comparison to the 20 canonical amino acids for protein, and specific base pairing in double stranded DNA is achieved via well-defined hydrogen-bonding interactions. Herein, we aim to show that an unnatural nucleoside (UNS), 4-cyanoindole-2′-deoxyribonucleoside (4CNI-NS) (Figure 1), can be used as a site-specific IR and fluorescence probe of DNA structure and dynamics.

The indole ring serves as a structural scaffold to the natural nucleobases adenine and guanine. Therefore, it is possible to use indole-based UNSs as universal DNA bases. Indeed, 5-nitroindole-2′-deoxyribonucleoside (5NI-NS) (Figure 1) has long been used as an unnatural universal DNA base, as it can pair with all four natural DNA bases [9]. More recently, Passow and Harki [10] have shown that 4CNI-NS can also be used as a universal DNA base. As demonstrated in previous studies [11,12,13], in comparison to indole and other indole derivatives, 4-cyanoindole (4CNI) (Figure 1) is significantly more fluorescent, has a long fluorescence lifetime, a red-shifted absorption spectrum, and an emission spectrum in the blue region of the visible spectrum. These qualities give 4CNI-NS a distinct advantage over 5NI-NS as it can serve as a fluorescence reporter. Moreover, the study of Passow and Harki [10] has shown that guanine can effectively quench the fluorescence of 4CNI-NS when in close proximity. This suggests that 4CNI-NS and guanine constitute a useful fluorophore-quencher pair for investigating various DNA-related questions via fluorescence spectroscopy, such as DNA-protein binding interaction, DNA–DNA association and conformational distribution of single-stranded DNA (ssDNA) in solution. To validate this notion, herein we carry out a proof-of-principle experiment in which we use fluorescence intensity measurements to determine the binding constant of a 4CNI-NS-containing ssDNA to a transport protein, bovine serum albumin (BSA).

While the IR spectrum of a DNA/RNA molecule manifests its structure and dynamics, it is hardly interpretable in a site-specific manner, due to spectral overlapping and degeneracy. Therefore, in order to achieve site-specificity in IR measurement of DNA/RNA, an appropriate UNS-based vibrational probe is needed. To the best of our knowledge, there is only one such UNS, i.e., a uridine derivative [14], that can be used in this purpose, hence limiting the use of IR spectroscopy to gain site-specific structural and dynamical information of DNA. To overcome this limitation, we first examine the utility of the universal base, 5NI-NS, as a site-specific IR probe. We find that while the nitro group of 5NI-NS gives rise to an intense band at ~1530 cm^−1^, its frequency is insensitive to a solvent, thus making it less useful as a vibrational probe. Several studies have shown that the C≡N stretching vibration of an alkyl or aryl nitrile is not only located at an uncongested region of the IR spectrum of bio-macromolecules, but is also sensitive to the local environment [15,16,17,18]. Hence, several nitrile-containing UAAs have been employed to site-specifically interrogate various properties of the protein system in question via linear and/or nonlinear IR methods [2,19]. These previous studies lead us to believe that the C≡N stretching vibration of 4CNI-NS can also be used as a site-specific IR probe of DNA structure and dynamics. To corroborate this notion, we examine the dependence of the C≡N stretching vibrational frequency (ν_CN_) of 4CNI-NS on a solvent. Our results show that ν_CN_ is sensitive to a solvent, similar to that observed for 4-cyanotryptohan (4CN-Trp) [13]. Therefore, this finding supports the idea that 4CNI-NS can be utilized to provide information about the local hydration and electrostatic environment of DNA in a site-specific manner.

## 2. Results and Discussion

### 2.1. Fluorescence Study

**Figure 2:**
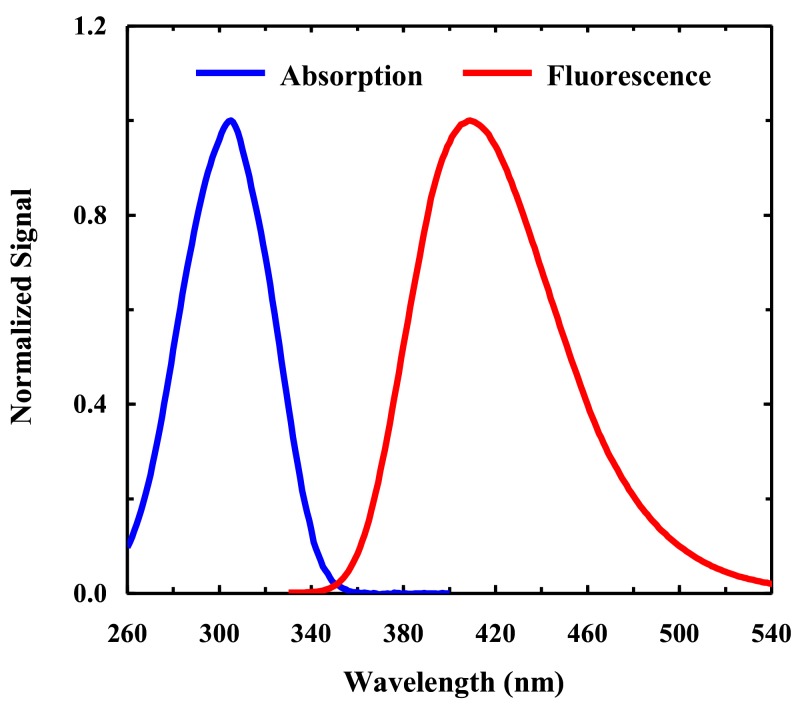
Normalized absorption and fluorescence spectra of 4-cyanoindole-2′-deoxyribonucleoside (4CNI-NS) (λ_ex_ = 320 nm) in water, as indicated.

The absorption spectrum of 4CNI-NS extends beyond 310 nm (Figure 2), indicating that its florescence can be selectively excited in the presence of aromatic amino acids. This feature is especially convenient for studies involving proteins, such as DNA–protein interactions. As shown (Figure 2), the fluorescence spectrum of 4CNI-NS peaks at ~412 nm in water, exhibiting a relatively large stokes shift. Furthermore, the fluorescence quantum yield (QY) of 4CNI-NS, determined using 4CNI as a reference, is 0.85 ± 0.5 in water, which is in agreement with that measured by Passow and Harki [10]. Since the fluorescence QY of 4CN-Trp is ~0.8 in water, this result indicates that covalently connecting a molecular group at either the 1 or 3 position of the 4CNI ring does not significantly change its fluorescence property. In addition, similar to that observed for the 4CN-Trp [12], in the hydrophobic solvent tetrahydrofuran (THF), the fluorescence QY of 4CNI-NS is decreased to 0.72, while the peak wavelength (λ_em_) of its fluorescence spectrum is blue-shifted to 380 nm. This blue-shift, similar to that observed for indole, is a manifestation of the less stabilizing effect of a less polar solvent on the fluorophore’s excited state, whose permanent dipole moment is different from the corresponding ground-state value [20]. These changes suggest that both λ_em_ and QY of 4CNI-NS fluorescence can potentially be useful as indictors of its local environment. However, given the fact that other nucleosides can quench the fluorescence of 4CNI-NS (see below), only λ_em_ is practically useful in this regard.

Interestingly, the study of Passow and Harki [10] demonstrated that the fluorescence of 4CNI-NS can be quenched by guanine. It has been shown that guanine can quench the fluorescence of various fluorescent dyes via the mechanism of electron transfer (ET) [21,22]. Based on those previous findings, we believe that the same quenching mechanism is also at play for 4CNI-NS. Since the ET transfer rate exhibits an exponential distance dependence [20], efficient fluorescence quenching via an ET mechanism can only occur when the corresponding fluorophore and quencher are sufficiently close or in Van der Waals contact [20,21,22]. This property thus makes 4CNI-NS and guanine a very useful fluorophore-quencher pair that can be used to study, for example, DNA-protein interactions. To demonstrate this utility, we employ it to determine the binding constant of a ssDNA to BSA via fluorescence spectroscopy. In practice, DNA–protein associations are typically detected by techniques based on ultra-centrifugation, isothermal titration calorimetry (ITC) or surface plasmon resonance (SPR) [23], which are time consuming and relatively low throughput. Therefore, devising a fluorescence-based assay would be quite advantageous, as it will provide a more convenient and potentially high-throughput means to explore specific DNA–protein binding interactions.

**Figure 3:**
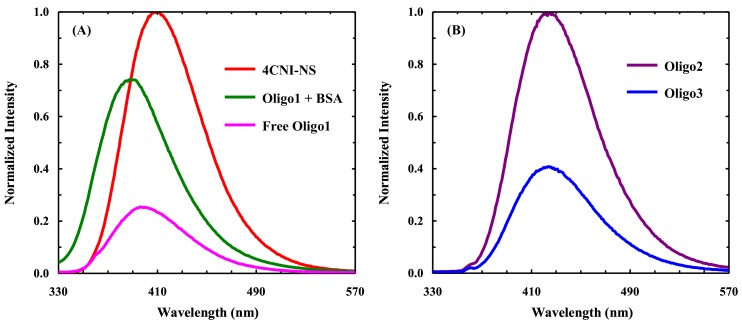
(**A**) Normalized fluorescence spectra of 4-cyanoindole-2′-deoxyribonucleoside (4CNI-NS), free Oligo1, and an Oligo1-BSA mixture, as indicated. (**B**) Normalized fluorescence spectra of Oligo2 and Oligo3, as indicated. These spectra were collected under the same experimental conditions (i.e., temperature, excitation/emission slit, and fluorophore absorbance at λ_ex_ = 320 nm).

BSA is an abundant carrier protein in blood that has been shown to promiscuously bind with DNA and RNA oligonucleotides amongst other molecules such as fatty acids, small molecules, drugs, and peptides [24]. To use fluorescence spectroscopy to probe the binding interaction between a ssDNA and BSA, we synthesized the following 18-base oligonucleotide (Oligo1): 5′-ACTTGGCC(4CNI-NS)CCAATTTTG. This sequence is designed with the consideration that a ssDNA molecule bound to a protein often adopts a more extended conformation in comparison to its free form [25]. Therefore, the fluorescence QY of Oligo1 is expected to increase due to the increase in the (average) separation distance between the 4CNI-NS fluorophore and the guanine quenchers upon binding to BSA. As shown (Figure 3A), the fluorescence spectra obtained under different solution conditions meets our expectation: (1) the fluorescence intensity of free Oligo1 is significantly smaller than that of 4CNI-NS, confirming the quenching effect of guanine; and (2) in the presence of BSA, the fluorescence spectrum of Oligo1 not only is blue-shifted (~8 nm) but also exhibits a larger intensity, which, combined, indicates protein binding.

To further validate the exclusive quenching effect of guanine toward the fluorescence of the 4CNI fluorophore in 4CNI-NS, we compared the fluorescence QYs of two other oligonucleotides. As indicated by their sequences, 5′-4CNI-ACTTAACCACCATTTTT (Oligo2) and 5′-4CNI-ACTTAACCGCCATTTTT (Oligo3), while each oligonucleotide has a 4CNI fluorophore appended at the 5′ end, only Oligo3 contains a guanine base. As shown (Figure 3B), under identical experimental conditions (i.e., concentration, excitation wavelength, solvent, temperature, and absorbance at λ_ex_) the fluorescence intensity of Oligo2 is larger than that of Oligo3. Consistent with this finding, a more quantitative assessment revealed that the fluorescence QYs of Oligo2 and Oligo3 are 0.74 and 0.31, respectively. Therefore, taken together, these results support our proposal that 4CNI-NS and guanine constitute a fluorophore-quencher pair useful for studying various questions in DNA science, similar to those used in protein science [26,27,28].

**Figure 4:**
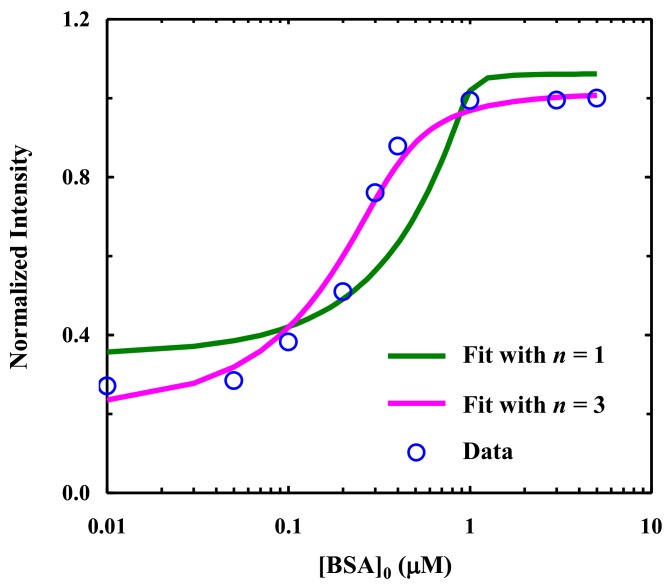
Normalized fluorescence intensity of Oligo1 (1.0 μM) as a function of the total concentration of BSA (open circles). The solid lines are best fits of these data to Equation (2) with different *n* values, as indicated. For *n* = 3, the fit yielded a *K*_d_ of 125 nM.

Finally, to demonstrate the utility of the proposed method, we employed fluorescence spectroscopy to determine the binding constant of Oligo1 to BSA. Specifically, we collected the fluorescence spectra of a series of solutions consisting of 1.0 μM Oligo1 and various concentrations of BSA ([BSA]) using an λ_ex_ of 320 nm. As indicated (Figure 4), the fluorescence intensity of the 4CNI-NS fluorophore in Oligo1 increases with increasing [BSA] and levels off at ~1 μM of BSA, indicating that Oligo1 has a strong affinity toward BSA. To provide a more quantitative assessment of this fluorescence binding curve, we analyzed it using a simple thermodynamic model that assumes that each BSA molecule can provide *n* identical, non-interacting binding sites. In other words, the effective concentration of BSA is scaled by *n*. It can be easily shown that:

(1) $$\left[D_{B}\right]=\frac{\left([D{]}_{0}+n[\mathrm{BSA}{]}_{0}+K_{d}\right)-\sqrt{{\left([D{]}_{0}+n[\mathrm{BSA}\right]}_{0}+K_{d}{)}^{2}-4n[D{]}_{0}[\mathrm{BSA}{]}_{0}}}{2}$$

where [*D*]_0_ and [BSA]_0_ are the initial or total concentrations of Oligo1 and BSA, respectively; [*D*_B_] is the concentration of BSA-bound Oligo1; and *K*_d_ is the dissociation constant. Since the fluorescence signal contains contributions from the free and BSA-bound Oligo1 molecules, the binding curve (i.e., *I* versus [BSA]_0_) is fit to the flowing equation:

(2) $$I=I_{F}\times \left([D{]}_{0}-[D_{B}\right])+I_{B}\times \left[D_{B}\right]$$

where *I*_F_ and *I*_B_ are the fluorescence intensities of the free and BSA-bound Oligo1 molecules, respectively, which, along with *K*_d_, were treated as fitting parameters. As shown (Figure 4), a single-binding-site model (i.e., *n* = 1) does not fit the experimental data well. Whereas the fluorescence binding curve can be fit reasonably well by a three-binding-site model (i.e., *n* = 3) with a *K*_d_ = 125 nM. While binding cooperativity is not considered in this simple model, the result is consistent with many studies showing that BSA can provide multiple binding sites for various ligands. In addition, the value of *K*_d_ is in the range of those determined for other DNA-protein systems, which are usually in the pM to nM range. Although BSA is not a specific DNA binder, this result indicates that it does have a relatively high affinity for ssDNA [24,25,29].

**Figure 5:**
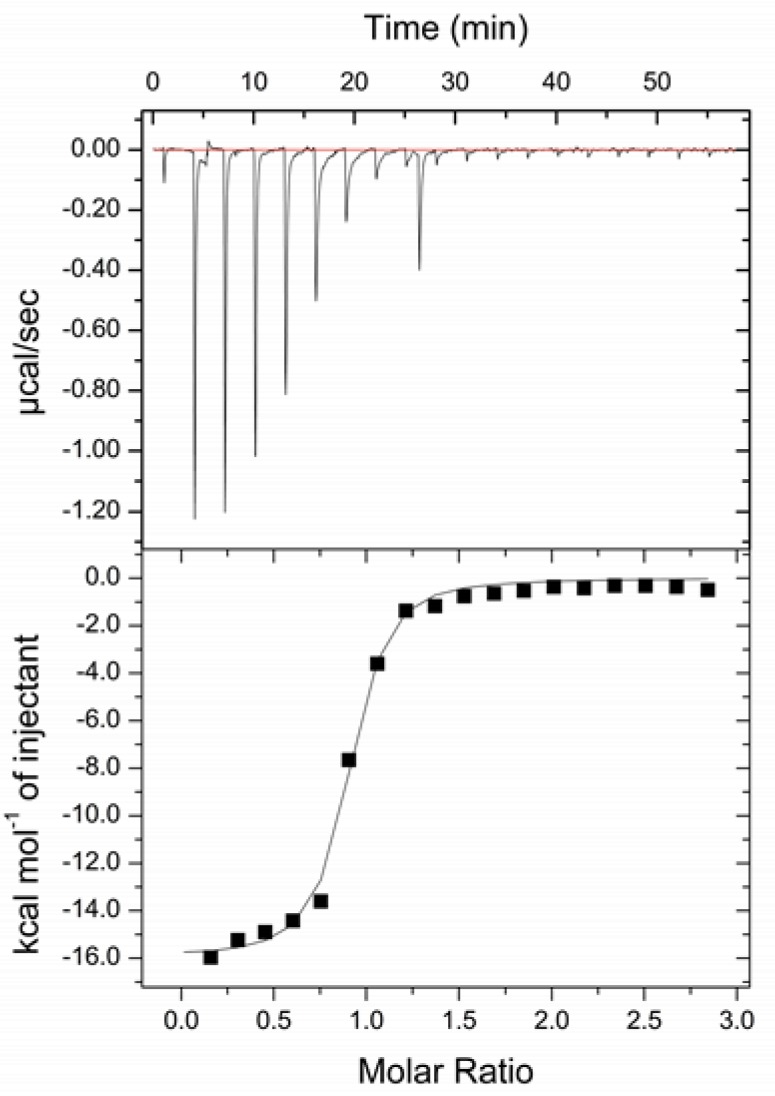
ITC heat signature (top panel) and the corresponding binding curve (bottom panel) when Oligo1* (30 μM) is titrated with BSA (300 μM). The solid line in the bottom panel is a guide to the eye.

To further validate this fluorescence binding assay, we used ITC to determine the BSA binding affinity of Oligo1*, whose sequence is identical to that of Oligo1 except that the 4CNI-NS base is replaced with guanine. As shown (Figure 5), the ITC measurements yielded a binding curve that is similar to that determined for Oligo1 via fluorescence spectroscopy. Therefore, this result not only corroborates the aforementioned fluorescence method, but also demonstrates that replacing a guanine base in a DNA with 4CNI-NS will not significantly affect its interaction with proteins.

### 2.2. FTIR Study

**Figure 6:**
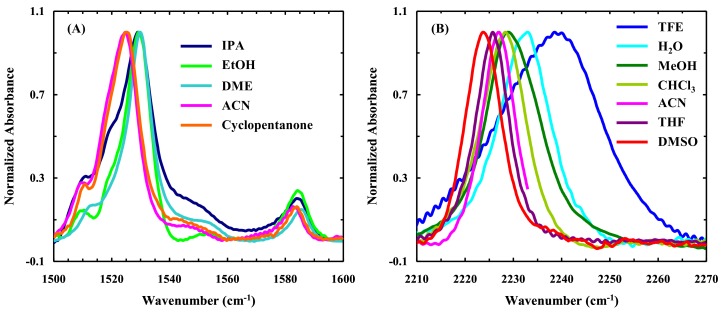
(**A**) FTIR spectra (in the region of the asymmetric stretching vibrational band of the nitro group) of 5-nitroindole (5NI) in isopropanol (IPA), ethanol (EtOH), dimethyl ether (DME), acetonitrile (ACN), and cyclopentanone, as indicated. (**B**) The C≡N stretching band of 4-cyanoindole-2′-deoxyribonucleoside (4CNI-NS) in different solvents, as indicated. Only part of the spectrum obtained in ACN is shown, due to the interference of the C≡N stretching band of the solvent at 2267 cm^−1^. For easy comparison, the original spectra, which have a maximum absorbance of ~10 mOD, have been normalized.

Identifying UNSs that have a unique vibrational mode that can be used as a site-specific IR probe of polynucleotides would open up new avenues in the study of DNA/RNA structure and dynamics using IR spectroscopy. Herein, we examine whether 5NI-NS and 4CNI-NS affords such utility. 5NI-NS is a widely-used universal base because of its ability to pair with all four nucleobases (A, T, C, G) through aromatic stacking. The asymmetric stretching frequency of the –NO_2_ (nitro) group in nitrobenzene is around 1550 cm^−1^ [30], suggesting that this vibrational mode of 5NI-NS could be useful as a site-specific IR probe. To verify this notion, we measured the FTIR spectra of 5-nitro-indole (5NI), the functional group of 5NI-NS, in different solvents. As shown (Figure 6A), in the spectral region of 1500–1600 cm^−1^, the FTIR spectrum of 5NI is rather complex and, perhaps more importantly, the asymmetric stretching frequency of the nitro group does not exhibit a simple dependence on the solvent. For example, the frequencies obtained in ethanol, a protic solvent with a dielectric constant of 24.5, and dimethyl ether, an aprotic solvent with a dielectric constant of 4.3, are nearly identical. In addition, polynucleotides containing A and G show intrinsic vibrational bands in this region of the spectrum [31,32]. These factors suggest that 5NI-NS is unlikely to be useful as a site-specific IR probe of DNA structure and dynamics.

In comparison, the C≡N stretching vibrational band of 4CNI-NS is simpler and exhibits a more sensitive solvent dependence (Figure 6B and Table 1). For example, its frequency is ~2232 cm^−1^ in water, which is shifted to ~2226 cm^−1^ in THF. This change is similar to that observed for *p*-cyanophenylalanine [15], a widely used site-specific IR probe of proteins, and 4CN-Trp [13]. Thus, these results, in conjunction with the fact that the C≡N stretching band is in an uncongested region of the IR spectrum of most natural bio-macromolecules [1,2], suggests that 4CNI-NS is a suitable site-specific IR probe of local hydration and electrostatic environment of DNA and RNA.

**Figure 7:**
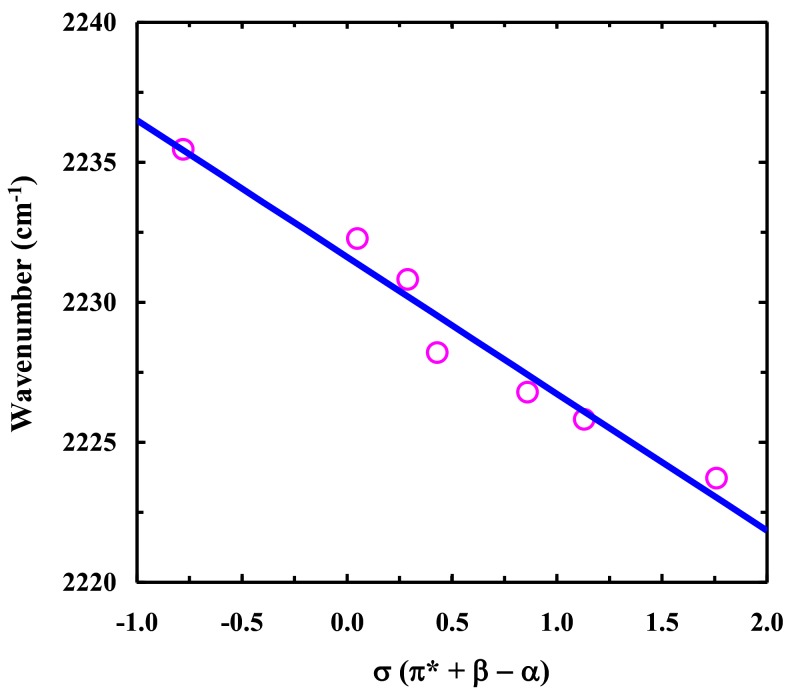
Center frequency of the C≡N stretching band of 4-cyanoindole-2′-deoxyribonucleoside (4CNI-NS) versus the solvent σ parameter. The solid line represents the linear regression of these data, yielding a slope of –4.9 cm^−1^ and an intercept of 2231.6 cm^−1^.

Zhang et al. [33] have shown that the C≡N stretching frequency of another cyanoindole (i.e., 3-methyl-5-cyanoindole) is linearly correlated with an empirical solvent parameter σ = π^*^ + β − α, where π^*^ (polarizability), β (hydrogen bond accepting ability), and α (hydrogen bond donating ability) are the Kamlet–Taft solvent parameters [34]. This linear relationship suggests that both specific interactions, i.e., hydrogen-bonding interactions (through α and β), and non-specific interactions (through π^*^) with the molecule work together to determine the C≡N stretching frequency of cyanoindoles. Because of the structural similarity between 4-cyanoindole and 5-cyanoindole, we expect that the C≡N stretching frequency of 4CNI-NS also shows a linear dependence on σ, as observed (Figure 7). Such a linear dependence is quite useful in practice, as it allows a more straightforward and quantitative interpretation of the result (according to the Kamlet–Taft treatment).

**Figure 8:**
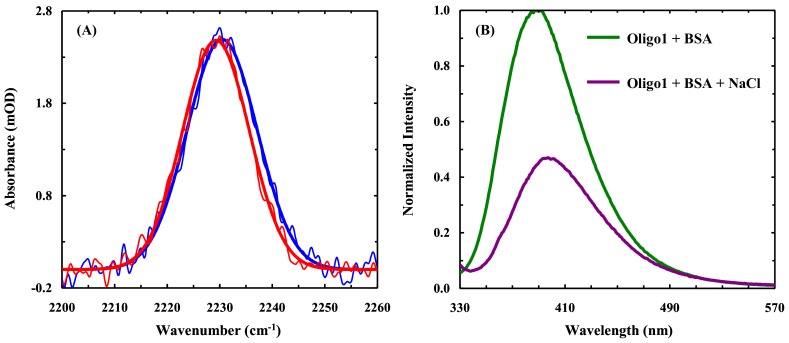
(**A**) Comparison of the C≡N stretching bands of Oligo1 obtained in the presence (red) and absence (blue) of BSA. **(B**) Normalized fluorescence spectra of an Oligo1-BSA mixture in the presence and absence of 2 M NaCl, as indicated.

As discussed above, the fluorescence spectrum of BSA-bound Oligo1 is only modestly blue-shifted (i.e., by ~8 nm). This suggests that the local electrostatic environment of the 4CNI-NS in Oligo1 does not change significantly upon BSA binding. Consistent with this picture, the C≡N stretching frequency of 4CNI-NS in Oligo1 is only red-shifted by ~1.2 cm^−1^, when bound to BSA (Figure 8A). Taken together, these results not only confirm the practical utility of the C≡N stretching mode of 4CNI-NS, but also suggest that the interaction between Oligo1 and BSA is electrostatic in nature. If the binding was mainly controlled by hydrophobic forces, one would expect to observe a much larger shift in both the IR and fluorescence spectra. Indeed, in support of this notion, the fluorescence intensity of 4CNI-NS is significantly decreased when 2 M NaCl is added to the Oligo1-BSA solution in question (Figure 8B), due to salt-induced dissociation of Oligo1 from the protein through the charge screening effect. Finally, it is worth noting that the C≡N stretching frequency of individual 4CNI-NS in water is ~2232.3 cm^−1^, whereas that of Oligo1 in water is ~2230.5 cm^−1^. This red-shift is consistent with the aforementioned notion that Oligo1 can adopt an ensemble of compact conformations, leaving the 4CNI-NS base partially dehydrated.

## 3. Materials and Methods 

### 3.1. Sample Preparation

Synthesis of 5′-O-dimethoxytrityl protected 4CNI-NS phosphoramidite building block was prepared according to a known literature method [10]. Oligo1 (5′-ACTTGGCC(4CNI-NS) NCCAATTTTG) was synthesized on an Applied Biosystems Expedite 8909 DNA synthesizer (Carlsbad, CA, USA) using a standard phosphoramidite method on 1 µmole scale as previously described [35]. A mild cleavage condition (saturated ammonium hydroxide solution overnight at room temperature) was employed to minimize the hydrolysis of the nitrile group. Oligo1* (5′-ACTTGGCCGNCCAATTTTG) was purchased from Integrated DNA Technologies (Coralville, IA, USA).

Oligo2 (5′-4CNI-ACTTAACCACCATTTTT) and Oligo3 (5′-4CNI-ACTTAACCGCCATTTTT) were generated by coupling 4-cyanoindole-3-acetic acid (4CNI-3AA) to 5′ amino modified oligonucleotides with 6-carbon linker which were purchased from Integrated DNA Technologies. Specifically, a solution of 4CNI-3AA (33 mg, 0.16 mmol) and sulfo-NHS (N-hydroxysuccinmide) (93 mg, 0.21 mmol) in anhydrous DMF (1 mL) was added a solution of DCC (44 mg, 0.21 mmol) in DMF (1 mL) at 0 °C. The mixture was stirred for 2 h under N_2_ and poured into a solution of oligonucleotide (3.7 µmol) in phosphate buffer (0.1 M, pH 7.2, 20 mL) and stirred overnight. All oligonucleotides were purified by reversed-phase high-performance liquid chromatography using Agilent 1100 HPLC system (Santa Clara, CA, USA) to achieve >95% purity. Mass spectrometry on a Shimadzu AXIMA Performance MALDI-TOF (Kyoto, Japan) was used to confirm product mass and purity. The concentrations of oligonucleotides and BSA (Sigma, St. Louis. MO, USA) were determined by UV/VIS (JASCO, Easton, MD, USA) using ε values of 7,790 M^−1^ cm^−1^ at 305 nm [7] and 43,824 M^−1^ cm^−1^ at 280 nm, respectively.

### 3.2. Fluorescence Measurement

All fluorescence spectra were collected on a Jobin Yvon Horiba Fluorolog 3.10 spectrofluorometer (Kyoto, Japan) at room temperature in a 1 cm quartz cuvette with a 1.0 nm resolution, 1 nm excitation/emission slit, an integration time of 1.0 nm/s, and an excitation wavelength of either 320 or 325 nm. All 4CNI-based samples were prepared by directly dissolving lyophilized solids in pure water (or THF), and the final concentration was 5.0 μM, except for that used in the BSA binding study, which was 1.0 μM. Fluorescence QY was determined using the following equation [22]:

(3) $$QY_{S}=\;QY_{R}\frac{I_{S}A_{R}}{I_{R}A_{S}}$$

where *I* is the integrated fluorescence intensity, *A* is the optical density of the fluorophore at λ_ex_ (325 nm), and the subscripts S and R represent the sample and reference, respectively. In the current study, 4CNI was used as the reference (*QY*_R_ = 0.78 in water) [12].

### 3.4. Isothermal Titration Calorimetry Measurement

ITC experiments were carried out on a MicroCal iTC200 (Malvern, UK), using the following instrument settings: 20 injections, initial delay 60 sec., spacing 180 sec., filter period 5 sec., injection volume 2 μL, measurement temperature of 25 °C, reference power of 6 μcal s^−1^, and stirring speed of 1000 r.p.m. The BSA concentration in the syringe was 300 μM and the Oligo1* concentration in the cell was 30 μM.

### 3.5. FTIR Measurement

FTIR spectra were collected on a Nicolet Magna-IR 860 spectrometer (ThermoFisher Scientific, Waltham, MA, USA) using a home-made sample holder composed of two CaF_2_ windows and a 50 μm spacer. All samples were prepared by directly dissolving lyophilized solids in the specified solvents with a final concentration of ~10 mM.

## 4. Conclusions

We find that the C≡N stretching frequency of 4CNI-NS, a universal nucleobase, is dependent on the solvent. Because of its sensitivity to the environment and the fact that this vibrational band is located in an uncongested region of the IR spectrum of biological macromolecules, it can be used as a site-specific vibrational probe to assess the local hydration and electrostatic environment of DNA and DNA–protein complexes. Furthermore, we devised a fluorescence assay, which relies on the quenching of 4CNI-NS fluorescence by guanine, for determining the binding constant of ssDNA–protein complexes. Since fluorescence measurement is easy, widely-available, low-cost, and can be performed in a high-throughput manner, we believe that this method will find wide application in the study of DNA–protein interactions. Moreover, it is our expectation that this fluorophore-quencher pair can find other novel applications. For example, it can be used (1) to study the kinetics of DNA–DNA interactions; (2) determine the conformational distribution of ssDNAs, similar to that done for peptides [36]; (3) characterize the rate of intermolecular contact formation of ssDNAs; (4) study the thermodynamics and kinetics of DNA/RNA folding; and (5) interrogate DNA base flipping dynamics, similar to that done by 2-aminopurine [37].

## Figures and Tables

**Table 1:** The center frequency (ω_0_) and full-width at half maximum (FWHM) of the C≡N stretching band of 4-cyanoindole-2′-deoxyribonucleoside (4CNI-NS) in different solvents. Also listed for each solvent are its Kamlet–Taft parameters, taken from Ref. [34].

Solvent	ω_0_, cm^−1^	FWHM, cm^−1^	π*	β	α	σ
Water (H_2_O)	2232.3	11.1	1.09	0.47	1.51	0.05
Methanol (MeOH)	2230.8	9.6	0.6	0.62	0.93	0.29
Dimethyl sulfoxide (DMSO)	2223.7	7.4	1	0.76	0	1.76
Acetonitrile (ACN)	2226.8	10.1	0.75	0.31	0.19	0.87
Tetrahydrofuran (THF)	2225.8	7.17	0.58	0.55	0	1.13
Trifluoroethanol (TFE)	2235.4	22.6	0.73	0	1.51	−0.78
Chloroform (CHCl_3_)	2228.2	11.2	0.53	0.1	0.2	0.43

## References

[1] Serrano A.L., Waegele M.M., Gai F. (2012). Spectroscopic studies of protein folding: Linear and nonlinear methods. Protein Sci..

[2] Ma J., Pazos I.M., Zhang W., Culik R.M., Gai F. (2015). Site-specific infrared probes of proteins. Annu. Rev. Phys. Chem..

[3] Haney C.M., Wissner R.F., Petersson E.J. (2015). Multiply labeling proteins for studies of folding and stability. Curr. Opin. Chem. Biol..

[4] Hanoian P., Liu C.T., Hammes-Schiffer S., Benkovic S. (2015). Perspectives on electrostatics and conformational motions in enzyme catalysis. Acc. Chem. Res..

[5] Boxer S.G. (2009). Stark realities. J. Phys. Chem. B.

[6] Ghosh A., Ostrander J.S., Zanni M.T. (2017). Watching proteins wiggle: Mapping structures with two-dimensional infrared spectroscopy. Chem. Rev..

[7] Błasiak B., Londergan C.H., Webb L.J., Cho M. (2017). Vibrational probes: From small molecule solvatochromism theory and experiments to applications in complex systems. Acc. Chem. Res..

[8] Adhikary R., Zimmermann J., Romesberg F.E. (2017). Transparent window vibrational probes for the characterization of proteins with high structural and temporal resolution. Chem. Rev..

[9] Loakes D., Brown D.M. (1994). 5-Nitroindole as an universal base analogue. Nucleic Acids Res..

[10] Passow K.T., Harki D.A. (2018). 4–Cyanoindole-2′-deoxyribonucleoside (4CIN): A universal fluorescent nucleoside analogue. Org. Lett..

[11] Hilaire M.R., Mukherjee D., Troxler T., Gai F. (2017). Solvent dependence of cyanoindole fluorescence lifetime. Chem. Phys. Lett..

[12] Hilaire M.R., Ahmed I.A., Lin C.-W., Jo H., DeGrado W.F., Gai F. (2017). Blue fluorescent amino acid for biological spectroscopy and microscopy. Proc. Natl. Acad. Sci. USA.

[13] Van Wilderen L.J.G.W., Brunst H., Gustmann H., Wachtveitl J., Broos J., Bredenbeck J. (2018). Cyano-tryptophans as dual infrared and fluorescence spectroscopic labels to assess structural dynamics in proteins. Phys. Chem. Chem. Phys..

[14] Schmitz A.J., Hogle D.G., Gai X.S., Fenlon E.E., Brewer S.H., Tucker M.J. (2016). Two-dimensional infrared study of vibrational coupling between azide and nitrile reporters in a RNA nucleoside. J. Phys. Chem. B.

[15] Getahun Z., Huang C.Y., Wang T., De León B., DeGrado W.F., Gai F. (2003). Using nitrile-derivatized amino acids as infrared probes of local environment. J. Am. Chem. Soc..

[16] Fafarman A.T., Webb L.J., Chuang J.I., Boxer S.G. (2006). Site-specific conversion of cysteine thiols into thiocyanate creates an IR probe for electric fields in proteins. J. Am. Chem. Soc..

[17] Waegele M.M., Tucker M.J., Gai F. (2009). 5-Cyanotryptophan as an infrared probe of local hydration status of proteins. Chem. Phys. Lett..

[18] Bagchi S., Fried S.D., Boxer S.G. (2012). A solvatochromic model calibrates nitriles’ vibrational frequencies to electrostatic fields. J. Am. Chem. Soc..

[19] Ding B., Hilaire M.R., Gai F. (2016). Infrared and fluorescence assessment of protein dynamics: From folding to function. J. Phys. Chem. B.

[20] Lakowicz J.R. (1999). Principles of Fluorescence Spectrscopy.

[21] Seidel C.A.M., Schulz A., Sauer M.H.M. (1996). Nucleobase-specific quenching of fluorescent dyes. 1. Nucleobase one-electron redox potentials and their correlation with static and dynamic quenching efficiencies. J. Phys. Chem..

[22] Heinlein T., Knemeyer J.-P., Piestert O., Sauer M. (2003). Photoinduced electron transfer between fluorescent dyes and guanosine residues in DNA-hairpins. J. Phys. Chem. B.

[23] Jing M., Bowser M.T. (2011). Methods for measuring aptamer-protein equilibria: A review. Anal. Chim. Acta.

[24] Peters T.J. (1985). Serum albumin: Advances in protein chemistry.

[25] Cattan D., Bourgoin D., Joly M. (1969). Change of conformation of DNA by association with proteins. Eur. J. Biochem..

[26] Taskent-Sezgin H., Marek P., Thomas R., Goldberg D., Chung J., Carrico I., Raleigh D.P. (2010). Modulation of *p*-cyanophenylalanine fluorescence by amino acid side chains and rational design of fluorescence probes of α-helix formation. Biochemistry.

[27] Goldberg J.M., Speight L.C., Fegley M.W., Petersson E.J. (2012). Minimalist probes for studying protein dynamics: Thioamide quenching of selectively excitable fluorescent amino acids. J. Am. Chem. Soc..

[28] Mintzer M.R., Troxler T., Gai F. (2015). *p*-Cyanophenylalanine and selenomethionine constitute a useful fluorophore-quencher pair for short distance measurements: Application to polyproline peptides. Phys. Chem. Chem. Phys..

[29] Peters T.J. (1996). All About Albumin: Biochemistry, Genetics and Medical Applications.

[30] Clarkson J., Smith W.E. (2003). A DFT analysis of the vibrational spectra of nitrobenzene. J. Mol. Struct..

[31] Seuvre A.M., Mathlouthi M. (1987). F.T.-I.R. spectra of oligo- and poly-nucleotides. Carbohydr. Res..

[32] Wood B.R. (2016). The importance of hydration and DNA conformation in interpreting infrared spectra of cells and tissues. Chem. Soc. Rev..

[33] Zhang W., Markiewicz B.N., Doerksen R.S., Smith A.B., Gai F. (2016). C≡N stretching vibration of 5-cyanotryptophan as an infrared probe of protein local environment: What determines its frequency?. Phys. Chem. Chem. Phys..

[34] Kamlet M.J., Abboud J.L.M., Abraham M.H., Taft R.W. (1983). Linear solvation energy relationships. 23. A comprehensive collection of the solvatochromic parameters, π*, α, and β, and some methods for simplifying the generalized solvatochromic equation. J. Org. Chem..

[35] Weber R.J., Liang S.I., Selden N.S., Desai T.A., Gartner Z.J. (2014). Efficient targeting of fatty-acid modified oligonucleotides to live cell membranes through stepwise assembly. Biomacromolecules.

[36] Tucker M.J., Oyola R., Gai F. (2005). Conformational distribution of a 14-residue peptide in solution: A fluorescence resonance energy transfer study. J. Phys. Chem. B.

[37] Holz B., Klimasauskasm S., Serva S., Weinhold E. (1998). 2-Aminopurine as a fluorescent probe for DNA base flipping by methyltransferases. Nucleic Acids Res..

